# Arsenic in Food and Drink

**Published:** 1901-01-19

**Authors:** 


### The Hospital

A Journal of

The Medical Sciences and Hospital Administration.

Vol. XXIX.—No. 747.] Edited by Sir Henry Burdett, K.C.B., and Solomon C. Smith, M.D., M.R.C.P. [January 19, 1901.

### ARSENIC IN FOOD AND DRINK.

The disclosures recently made with regard to the presence of arsenic in the beer of certain breweries, and with regard to the sickness and mortality which it has occasioned, have produced upon the public mind effects which do not seem likely to be transitory, and which are also influencing, in different methods and degrees, the minds of politicians. The *Times* of Saturday contained two interesting contributions to the question, in the form of the report of a speech by Mr. Long, the President of the Local Government Board, and so far the official guardian of the public health, and of a letter from Mr. Chaplin, who held that office in Lord Salisbury's last administration, and who is believed not to have been entirely in accord with his chief on the question of relinquishing it. Mr. Chaplin is advocating the passage during the next session of Parliament of some form of "Pure Beer Bill," and deprecates the large reference made to the Royal Commission appointed to consider "the amount of recent exceptional sickness and death in England and Wales, attributable to poisoning by arsenic in beer or in other articles of food or drink." He contends that the concluding words will afford occupation to the Commission for a period of from five to seven years, and will have the indirect effect of indefinitely postponing legislation on the question of beer alone. His action in the matter is avowedly taken not merely in the interests of the consumer of beer, but also in the interests of agriculture, under the belief that a change in the law affecting brewers would be calculated to increase the demand for British barley, and with it the prosperity of farmers; while at the same time it would not be in absolute contradiction to our knowledge of human nature to suggest that the right honourable gentleman may not be entirely indifferent to an opportunity of occasioning some embarrassment to a ministry of which he is no longer a member. Mr. Long, on the other hand, calls attention to certain financial difficulties which stand in the way of hasty legislation, and which are the more worthy of consideration since he at the same time declared that, "If it was a question of public health, taxation would have to stand on one side altogether. That was a matter for which he was responsible and which he would take care of." Coming from Mr. Long, whose resolute disregard of ignorant clamour in relation to rabies has been of such great service to the country, these words are of no small significance, and may fairly be accepted as a pledge that he would not continue in office if measures which he thought necessary for the protection of the public were refused by his colleagues. In such circumstances there can be no occasion for panic; more especially as, in the immediate future, beer will probably be less exposed to arsenical contamination than any other article of consumption. Everybody concerned in either making, distributing, or swallowing it will be likely to demand a written or verbal warranty from his immediate predecessor in the transaction. Mr. Long pointed out that the repeal of the Malt Tax by Mr. Gladstone, and the licence then given to brewers to make beer as they pleased, "provided that it was wholesome and fulfilled certain conditions," was, in fact, a bargain between the trade and the Exchequer under which the latter obtained an annual revenue of nearly twelve millions from the finished beverage. His own function, moreover, as guardian of the public health, was not merely to protect that health against poisonous beer, but against poisonous food or drink of any description; and the glucose, from which most of the arsenic in beer appeared to have been derived, was extensively used in the manufacture of sweetmeats, confectionery, jams, and other articles, with regard to all of which protection was required; so that an Act affecting beer alone would be inadequate for the needs of his department. The article commonly sold under the name of "jam" would, we presume, be in especial need of supervision. Jam is defined by Dr. Johnson to be "a conserve of fruits boiled with sugar and water." We suspect that his knowledge of housekeeping was defective, and that the mention of water was due, as he himself said of another error, to "pure ignorance;" but, however, this may be, it is certain that since jam-making ceased to be a domestic art and became a manufacture, much of the stuff sold under the name has ceased to agree with any known definition. We lately heard of an instance in which a passer-by was induced, by a placard in a grocer's window, to buy what purported to be "apricot jam," and found it to consist largely of glucose and inferior gelatin. In this case the purchaser compelled the vendor, by a threat of prosecution, to fetch away the mess and to return the money paid for it; and was amused to find, a few days later, that the word

"compôte" had been substituted for "jam" on the placard. It may be presumed that the manufacturer had been communicated with, and that he was unable to substantiate the original description. Nothing is more probable than that a maker of arsenical glucose with a stock on hand no longer saleable to brewers would endeavour to dispose of it at a reduction to jam makers, in the hope that the quantity consumed at any one time would be too small to produce any deleterious effect. This hope might very possibly be justified by the event, inasmuch as the ordinary capacity for swallowing beer is greater than that for swallowing so-called "jam." Experience has shown, nevertheless, that the Local Government Board can not trust the safety of the lieges to so frail a reed as the honesty or conscience of a manufacturer; and it would be a boon to the public if legislation enabled them to procure "jam" when they asked and paid for it, leaving manufacturers at full liberty to prepare and sell any kind of non-poisonous "compôte" under a proper description. Mr. Long's words seem to point in this direction, and we shall be glad to see them justified by the event. It is essential that it should be understood that the question as it now stands is far too large to be covered by the common head-line " Arsenic in Beer," or to be met by legislation bearing upon beer alone.

